# Protein-protein interactions between RUNX3 and ZEB1 in chronic lung injury induced by methamphetamine abuse

**DOI:** 10.3389/fphar.2022.1025922

**Published:** 2022-11-21

**Authors:** Ning Bao, Lin Cheng, Yun Wang, Zhe Peng, Zhengkun Wang, Shuangquan Chen

**Affiliations:** ^1^ Department of Anesthesiology, Affiliated Foshan Maternity & Child Healthcare Hospital, Southern Medical University, Foshan, Guangdong, China; ^2^ Department of Pharmacy, Shenzhen People’s Hospital (The Second Clinical Medical College, Jinan University, The First Affiliated Hospital, Southern University of Science and Technology), Shenzhen, Guangdong, China; ^3^ Department of Clinical Pharmacology, School of Pharmacy, China Medical University, Shenyang, Liaoning, China

**Keywords:** methamphetamine, protein-protein interactions, RUNX3, Zeb1, lung injury

## Abstract

Methamphetamine (MA) is the most common and highly addictive substance abuse drug. Runt-related transcription factor 3 (RUNX3) and Zinc finger E-box-binding homeobox 1 (ZEB1) are associated with lung inflammation and fibrosis. However, the protein-protein interactions (PPIs) between RUNX3 and ZEB1 and its involvement in MA-induced chronic lung injury is still unclear. In this study, we evaluated lung injury using echocardiography, hematoxylin and eosin staining, and western blot analysis. The viability of alveolar epithelial cells (AECs) was assessed using cell counting kit-8. Molecular Operating Environment software, Search Tool for the Retrieval of Interacting Genes/Proteins database, co-immunoprecipitation, assay and confocal immunofluorescence assay were used to predict and identify the PPIs between RUNX3 and ZEB1. The expression of RUNX3 and ZEB1 were knockdown in AECs using siRNA. The results revealed that MA exposure increased the peak blood flow velocity of the pulmonary artery and the acceleration time of pulmonary artery blood flow. Further, exposure to MA also causes adhesion and fusion of the alveolar walls and altered AEC activity. A decrease in the expression of RUNX3 and an increase in the expression of ZEB1 and its downstream signaling molecules were observed on MA exposure. The PPIs between RUNX3 and ZEB1 were identified. Further, an increase in the protein binding rate of RUNX3-ZEB1 was observed in MA-induced lung injury. These results show interactions between RUNX3 and ZEB1. RUNX3 protects against lung injury; however, ZEB1 expression and the PPIs between ZEB1 and RUNX3 has deleterious effects on chronic lung injury induced by MA exposure. Our results provide a new therapeutic approach for the treatment of chronic lung injury due to MA exposure.

## 1 Introduction

Drug abuse is a global health concern. As per analysis conducted by National Surveys on Drug Use and Health, an estimated rate of methamphetamine (MA) abuse in adults was 0.66% (Jones et al., 2020). The primary cause of MA-associated mortality is lung and heart diseases, excluding overdose and accidental death (Xiaoshan et al., 2020). MA exposure induces pulmonary edema, pneumorrhagia, pneumothorax, pulmonary artery hypertension, etc. (Kevil et al., 2019). Various proteins, such as runt-related transcription factor 3 (RUNX3), are associated with MA-induced lung toxicity. RUNX3 alters reactive oxygen species (ROS) levels in chronic lung injury due to MA exposure (Shi et al., 2020). RUNX3 belongs to the RUNX family and has a highly conserved DNA sequence which plays an important role in various biological processes, like the development of the nervous system and aging (Kulkarni et al., 2018; Mevel et al., 2019). RUNX3 interacts with the proteins in the Hippo signaling pathway and suppresses yes-associated protein-mediated epithelial-mesenchymal transition (EMT), inflammation, stemness, and metastasis of the cells (Kulkarni et al., 2018). A previous study has demonstrated the involvement of RUNX3 in the development and homeostatic maintenance of the body (Yokomizo-Nakano and Sashida, 2021). Further, an increase in RUNX3 level enhances the sensitivity to radiotherapy in lung cancer patients (Gao et al., 2021). RUNX3 suppresses apoptosis of cells in the acute lung injury caused by pancreatitis. A previous study has demonstrated that RUNX3 is essential for the EMT of cells in chronic pulmonary damage due to MA (Shi et al., 2020). However, the role of RUNX3 in chronic lung injury caused by MA exposure is still unclear.

Zinc finger E-box-binding homeobox 1 (ZEB1) is a transcription factor that induces EMT and coordinates with other transcription factors to promote metastasis, invasion, and chemoresistance in tumors (Zhang et al., 2015; Cheng et al., 2021). ZEB1 interacts with the proteins of Hippo signaling pathway to promote the development of various solid tumors (Lehmann et al., 2016). ZEB1 induces stemness and chemoresistance in cancer cells and leads to the fibrosis of cells (Zhang et al., 2015). ZEB1 overexpression in hypertrophic alveolar epithelial cells (AECs) induces the EMT of alveolar epithelial type II cells (Yao et al., 2019). However, the involvement of ZEB1 in chronic lung injury caused by MA abuse is unknown.

To check if RUNX3 and ZEB1 are correlated, we hypothesized possible protein-protein interactions (PPIs) between RUNX3 and ZEB1. PPIs play an important role in various biological processes (Li B et al., 2019). A previous report suggests that PPIs between N-cadherin and Nectin-2 form the basis of cell-cell adhesion (Duraivelan et al., 2018). The PPIs between Amyloid precursor protein and Munc18-interacting proteins induce amyloid-β formation in Alzheimer’s disease (Bartling et al., 2021). With recent development in pharmaceuticals, various novel drugs targeting PPIs are currently in the pre-clinical and clinical stages. For example, 1-benzoyl 4-phenoxypiperidines, a small molecular inhibitor, blocks the PPIs between β-catenin and B-cell lymphoma 9 (Li et al., 2021). A previous study has shown that the PPIs between B-cell lymphoma-2 and Parkin mediate mitophagy *via* the PTEN-induced putative kinase 1/Parkin pathway in the lipopolysaccharide-induced lung injury (Zhang Z et al., 2020). However, the PPIs between RUNX3 and ZEB1 have never been reported. Further, the involvement of PPI between RUNX3 and ZEB1 in the chronic lung injury induced by MA is still obscure.

Based on these findings, our study aimed to investigate if long-term exposure to MA can influence the functions of RUNX3 and ZEB1. Further, we investigated the interactions between RUNX3 and ZEB1 and the involvement of the interactions between RUNX3 and ZEB1 in chronic lung injury induced by long-term exposure to MA.

## 2 Experimental procedures

### 2.1 Establishment of animal models

Thirty male BALB/C mice were obtained from SPF Biotechnology Co., Ltd (license number: SCXK 2019–0010, Beijing). The mice were randomly divided into the control and the MA groups. 1 mg/kg MA (China Criminal Police University, China) was injected intraperitoneally to the mice in the 1st week. Further, the weekly dose was increased by 1 mg/kg/week and was increased to 6 mg/kg by the 6th week (Zhang Y et al., 2020). The mice in the control group were injected an equivalent of 0.9% saline intraperitoneally. The mice were weighed daily. The percentage of weight gain in each group was calculated every week as per Formula 1. After 6 weeks, the right ventricular index (RVI) was calculated using Formula 2 to evaluate the remodeling of the right ventricle due to chronic pulmonary dysfunction caused by MA exposure (Wang et al., 2020). All experimental procedures involving animals were in compliance with the China Medical University Institutional Animal Care and Use Committee (IACUC Issue No. CMU2019215).

$$Percentage\;of\;the\;weight\;gain=\frac{average\;of\;the\;weekly\;weight-average\;of\;the\;initial\;weight}{average\;of\;the\;initial\;weight\;}\times 100\%$$ (1)

$$RVI=\frac{the\;weight\;of\;the\;right\;heart}{the\;weight\;of\;the\;left\;heart+the\;weight\;of\;the\;ventricular\;septum}$$ (2)

### 2.2 Echocardiography

Transthoracic echocardiography was performed using a VEVO (Visualsonics 3100) system to measure the cardiopulmonary function under chronic exposure to MA. The peak blood flow velocity of the pulmonary artery (PV), the acceleration time of pulmonary artery blood flow (PAT), and the right ventricular free wall thickness (RVWT) were measured.

Briefly, Mice were anesthetized using 1–2% isoflurane in air. To determine the RVWT, the M-mode probe was oriented to the long-axis tangent plane, which was adjacent to the breastbone and sloped towards the right shoulder of mice. The pulsed-wave doppler was used to measure PV and PAT. The pulsed-wave doppler probe was located at the long-axis in the vicinity of the breastbone. The angle of the probe was ≤60°, and the frequency was 24 MHz.

### 2.3 Hematoxylin and eosin staining

The lungs were infused with 0.9% physiological saline and were immersed in the fixative solution for 14 days. The lungs were then embedded in paraffin. The paraffin-embedded lung tissues were sectioned in 4 µm thick sections, and HE staining was performed per the manufacturer’s instruction (Solarbio Cat# G1120). To check for lung injuries, three fields on the section were randomly selected and analyzed (magnification: ×200, ×400).

### 2.4 Cell culture, treatment, and transfection

AECs (A549, Beijing Dingguo Changsheng Biotechnology Co., Ltd.) were cultured in Dulbecco’s Modified Eagle Medium (DMEM)F12 medium (Sevenbio, Beijing, China) supplemented with 10% fetal bovine serum and 1% penicillin/streptomycin at 37°C in 5% CO_2_. AECs were treated with 0.1, 0.5, 1, and 5 mM MA for 12, 24, and 48 h.

The siRNA sequence used for transfection is shown in Table 1. The transfections were performed using the Lipofectamine™ 8000 Transfection Reagent (Beyotime, Cat# C0533) according to the manufacturer’s instructions. The cells were treated with 5 mM MA at the time of highest transfection efficiency for 48 h. Further, western blot analysis and CCK-8 assay were performed for subsequent analysis.

**TABLE 1:** Base pairs of siRNAs for transfection.

	Sense	Antisense	Source
RUNX3	5′-CCC​UGA​CCA​UCA​CUG​UGU​UTT-3′	3′-AAC​ACA​GUG​AUG​GUC​AGG​GTT-5′	GENERAL BIOL
ZEB1	5′-GUU​CCA​AGU​UGC​UUC​ATA​UAT-3′	3′-TTC​CAA​GGG​GUU​ACA​UAA​UAU-5’	GENERAL BIOL
Negative control	5′-UUC​UCC​GAA​CGU​GUC​ACG​UTT-3′	3′-ACG​UGA​CAC​GUU​CGG​AGA​ATT-5′	Sangon Biotech

### 2.5 Cell counting kit-8 assay

The cells were allowed to grow until 70% confluency and then were digested with pancreatic enzymes. The cells were centrifuged, the supernatant was discarded, and the cell pellet was evenly diluted with 1 ml culture medium. The cells were evenly seeded in 96 well plates (Jet Biofil), and the cell viability was determined using the CCK-8 assay kit (APExBIO Cat# K1018) according to the manufacturer’s instructions.

### 2.6 Bio-informatics prediction

Search Tool for the Retrieval of Interacting Genes/Protein (STRING) database was used to generate the gene network associated with RUNX3 and ZEB1. The structure of RUNX3 was predicted by the Robetta server, and the structure of ZEB1 was predicted using the I-TASSER server. Molecular Operating Environment (MOE) software (CloudScientific Technology Co., Ltd) was used to predict the PPIs and analyze the binding sites between RUNX3 and ZEB1.

### 2.7 Co-Immunoprecipitation assay

The beads (Protein A/G PLUS-Agarose; Santa Cruz Biotechnology, Cat# sc-2003) were added to the AECs. The overall protein amount in each group was adjusted to the same level. The lysate was retained at 40 μL each as input. The beads were added to the rest of the lysate and were incubated at 4°C for 1 h with rotation, followed by centrifugation. The supernatant was mixed with beads and antibodies and incubated at 4°C overnight with rotation, followed by centrifugation. The supernatant was then discarded. The beads were washed, and a 2× SDS-PAGE sample loading buffer was added to the beads and boiled at 100°C for 5 min for western blot analysis. The protein binding rate of RUNX3-ZEB1 was calculated using Formula 3.

$$Protein\;binding\;rate\;of\;RUNX3-ZEB1=\frac{the\;protein\;density\;of\;RUNX3\;in\;IP\;group\left(CON/MA\right)}{the\;protein\;density\;of\;RUNX3\;in\;Input\;group\left(CON/MA\right)}$$ (3)

### 2.8 Confocal immunofluorescence assay

The cells were fixed, permeabilized, and incubated with the primary antibodies at 4°C overnight. The details of the antibodies are in Table 1. The Cy3-conjugated AffiniPure goat anti-mouse IgG (H + L, Proteintech, Cat# SA00009-1) and Fluorescein (FITC)-conjugated AffiniPure goat Anti-Mouse IgG (H + L, Proteintech, Cat# SA00003-1) were used for the immunofluorescence. All the specimens were observed under the oil immersion lens of a confocal laser scanning microscope (OLYMPUS, FV1000S-SIM/IX81) at ×800 magnification.

### 2.9 Western blot analysis

The proteins were separated by sodium dodecyl sulfate-polyacrylamide gel electrophoresis and transferred onto a polyvinylidene fluoride (PVDF) membrane. The PVDF membrane was incubated with corresponding primary antibodies (Table 2) at 4°C overnight. On the next day, the membranes were incubated with secondary antibodies [HRP-conjugated AffiniPure Goat Anti-Rabbit IgG (H + L), Proteintech, Cat# SA00001-2; HRP-conjugated AffiniPure Goat Anti-Mouse IgG (H + L), Proteintech, Cat# SA00001-1] for 2 h at room temperature. The membranes were visualized by DNR Bio-Imaging systems, and densitometric analysis was performed using ImageJ software.

**TABLE 2:** Primary antibodies for western blot analysis.

Primary antibodies	Dilution	Company	Catalogue
CTGF	1: 2000	ABclonal	A11067
Caspase-3	1: 2000	ABclonal	A11953
Cleaved Caspase-3	1: 2000	ABclonal	A11953
E-cadherin	1: 2000	Affinity	BF0219
IL-6	1: 2000	ABclonal	A0286
IL-1β	1: 1000	ABclonal	A1112
ICAM1	1: 2000	ABclonal	A5597
N-cadherin	1: 2000	ABclonal	A19083
Vimentin	1: 50000	Proteintech	60330-1-lg
PCNA	1: 2000	Proteintech	10205-2-AP
RUNX3	1: 1000	Cell Signaling Technology	13089
ZEB1	1: 2000	Proteintech	21544-1-AP
β-actin	1: 10000	Proteintech	66009-1-lg

### 2.10 Statistical analysis

The data were expressed as mean ± standard deviation. Statistical analysis was performed using GraphPad Prism 8.0.2. Student’s t-test, one-way analysis of variance (ANOVA), and two-way ANOVA were used for statistical comparisons. ANOVA was followed by Tukey multiple comparisons. *p* < 0.05 was considered statistically significant.

## 3 Results

### 3.1 Lung injury induced by chronic exposure to MA

#### 3.1.1 Hemodynamic analysis using transthoracic echocardiography

**FIGURE 1:**
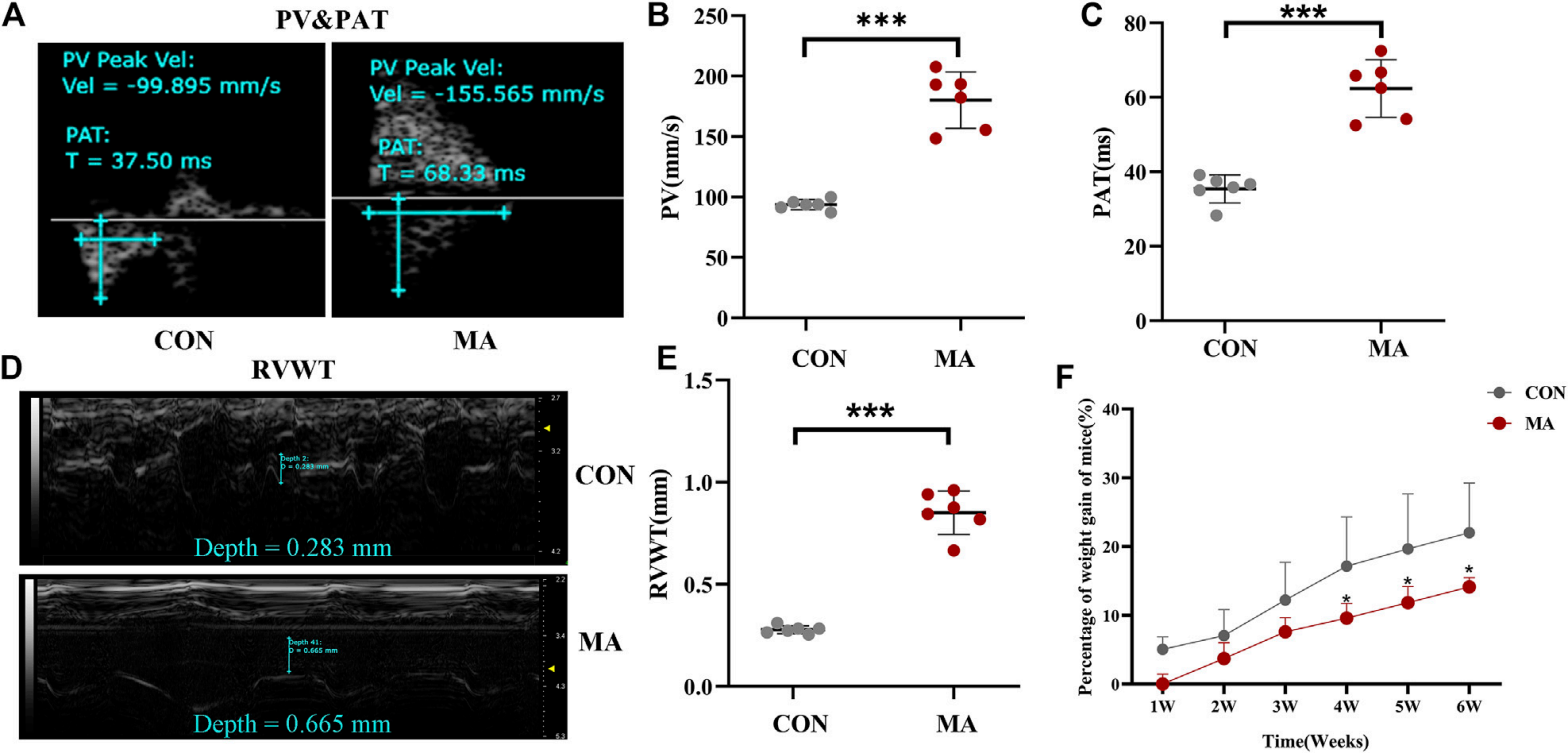
Hemodynamic analysis using transthoracic echocardiography and percentage of weight gain of mice. **(A)** The echocardiographic measurements for PV and PAT. **(B)** The PV in different groups. **(C)** The PAT in different groups. **(D)** The echocardiographic measurements for RV. **(E)** The echocardiographic measurements of RVWT in different groups. **(F)** Percentage of weight gain in the mice. N = 6. Data are expressed as mean ± standard deviation, ^*^ *p* < 0.05 vs. CON, ^**^ *p* < 0.01 vs. CON, ^***^ *p* < 0.001 vs. CON. PV: Peak blood flow velocity of the pulmonary artery, PAT: the acceleration time of pulmonary artery blood flow. RVWT: Right ventricular free wall thickness, CON: The control group, MA: mice treated with methamphetamine.

Transthoracic echocardiography was used to evaluate cardiopulmonary function, The pulmonary arterial pressure accelerated due to chronic lung injury induced by long-term exposure to MA (Savai et al., 2014). The PV (180.073 ± 23.261 mm/s) in the MA group was significantly higher compared to the PV (93.630 ± 4.224 mm/s) in the control group (^***^ *p* < 0.001 vs. CON; Figures 1A,B). As compared to the control group (35.417 ± 3.757 ms), a significant increase in the PAT (62.362 ± 7.717 ms) was observed in the MA group (^***^ *p* < 0.001 vs. CON; Figures 1A,C). The RVWT (0.851 ± 0.106 mm) in the MA group was significantly thicker compared to the RVWT (0.278 ± 0.019 mm) in the control group (^***^ *p* < 0.001 vs. CON; Figures 1D,E). Moreover, the percentage of weight gain in mice in the MA group was significantly slower compared to mice in the control group from the 4^th^ week (CON: 17.152 ± 7.161%; MA: 9.58 ± 2.148%) to the 6^th^ week of MA exposure (CON: 22 ± 7.238%; MA: 14.14 ± 1.318%, ^*^ *p* < 0.05 vs. CON; Figure 1F).

#### 3.1.2 Alveoli injury induced by MA exposure

**FIGURE 2:**
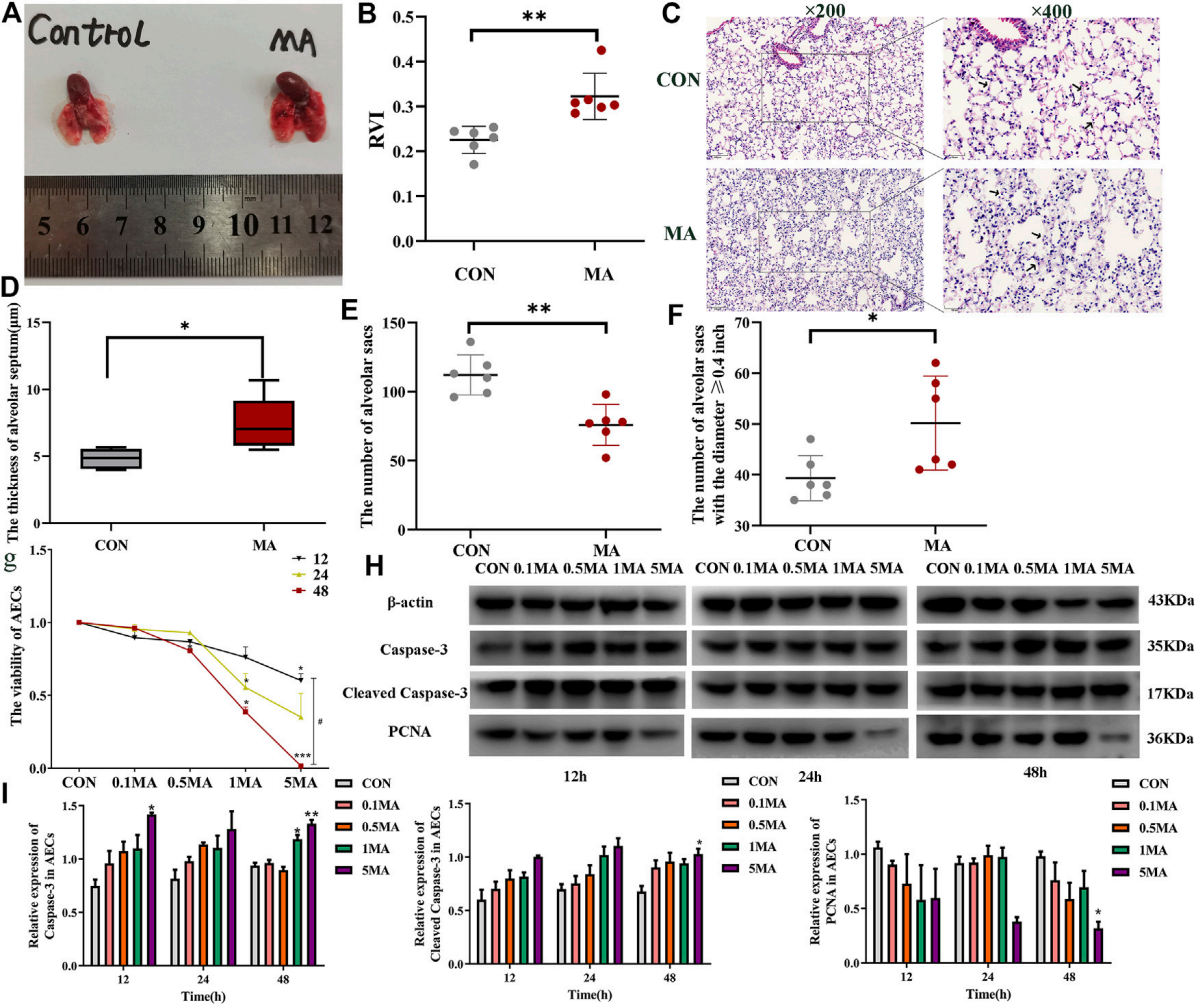
MA induced chronic lung injury. **(A)** Mice lungs in the CON group and the MA group. **(B)** RVI in different groups. **(C)** Morphological analysis of the mice lungs by HE staining. Magnification: ×200, ×400. The black arrows indicate the normal lung tissues in the CON group and the damaged lung tissues in the MA group. **(D)** The thickness of the alveolar septum in the mice lung, n = 6. **(E)** Number of alveolar sacs in the lung of the mice in the CON and the MA groups, n = 6. **(F)** Number of alveolar sacs in the lung of the mice in the CON and the MA groups with the diameter ≥0.4 inches, n = 6. **(G)** Viability of AECs on 0.1 mM, 0.5 mM, 1 mM, and 5 mM MA exposure after 12 h, 24 h, and 48 h using CCK-8 assay, n = 3. **(H**,**I)** Effect of different dosages and exposure time of MA on caspase-3, cleaved caspase-3 and PCNA expression was detected by western blots, n = 3. Data are expressed as mean ± standard deviation, ^*^ *p* < 0.05 vs. CON, ^**^ *p* < 0.01 vs. CON, ^***^ *p* < 0.001 vs. CON; ^#^ *p* < 0.05 vs. 5MA/12 h. RVI: Right ventricular index, AECs: Alveolar epithelial cells, CON: The control group, MA: The group treated with methamphetamine in mice, 0.1 MA: 0.1 mM methamphetamine, 0.5 MA: 0.5 mM methamphetamine, 1 MA: 1 mM methamphetamine, 5 MA: 5 mM methamphetamine.

The mice’s lungs in the MA group were inflamed and had redness compared to the lungs of mice in the control group (Figure 2A). The RVI (0.323 ± 0.051) in the MA group was significantly higher compared to the RVI (0.226 ± 0.003) in the control group, indicative of the right ventricular remodeling due to MA exposure-induced chronic lung injury (^**^ *p* < 0.01 vs. CON; Figure 2B) (Wang et al., 2020). HE staining results reveal that the alveolar walls were thin and delicate in the control group. In the MA group, the alveolar fusion was larger, and the alveolar walls were thicker compared to the control group, as indicated by black arrows in Figures 2C,D (^*^ *p* < 0.05 vs. CON). The sum of pulmonary alveoli in the MA group was less compared to the control group (^**^ *p* < 0.01 vs. CON; Figure 2E). In the MA group, the number of augmented pulmonary alveoli with a diameter ≥0.4 inches was more compared to the control group (^*^ *p* < 0.05 vs. CON; Figure 2F).

#### 3.1.3 AECs apoptosis induced by MA exposure

We treated AECs with 0.1, 0.5, 1 and 5 mM MA for 12, 24 and 48 h (Shi et al., 2020; Wang et al., 2020). The CCK-8 assay results revealed that the viability of AECs on MA exposure was time and dosage-dependent (^*^ *p* < 0.05 vs. CON, ^***^ *p* < 0.001 vs. CON, ^#^ *p* < 0.05 vs. 5 MA/12 h; Figure 2G). To investigate if apoptosis of AECs was induced by MA exposure, the expression of apoptosis proteins like caspase-3, cleaved caspase-3 and proliferating cell nuclear antigen (PCNA) were detected by western blot analysis. In AEC treated with MA, an increase in expression of caspase-3 and cleaved caspase-3 was observed in a time- and dosage-dependent manner (^*^ *p* < 0.05 vs. CON, ^**^ *p* < 0.01 vs. CON; Figures 2H,I). The expression of PCNA significantly decreased in the MA group compared to the control group (^*^ *p* < 0.05 vs. CON; Figures 2H,I).

#### 3.1.4 Inflammatory and fibrotic changes induced by MA exposure

**FIGURE 3:**
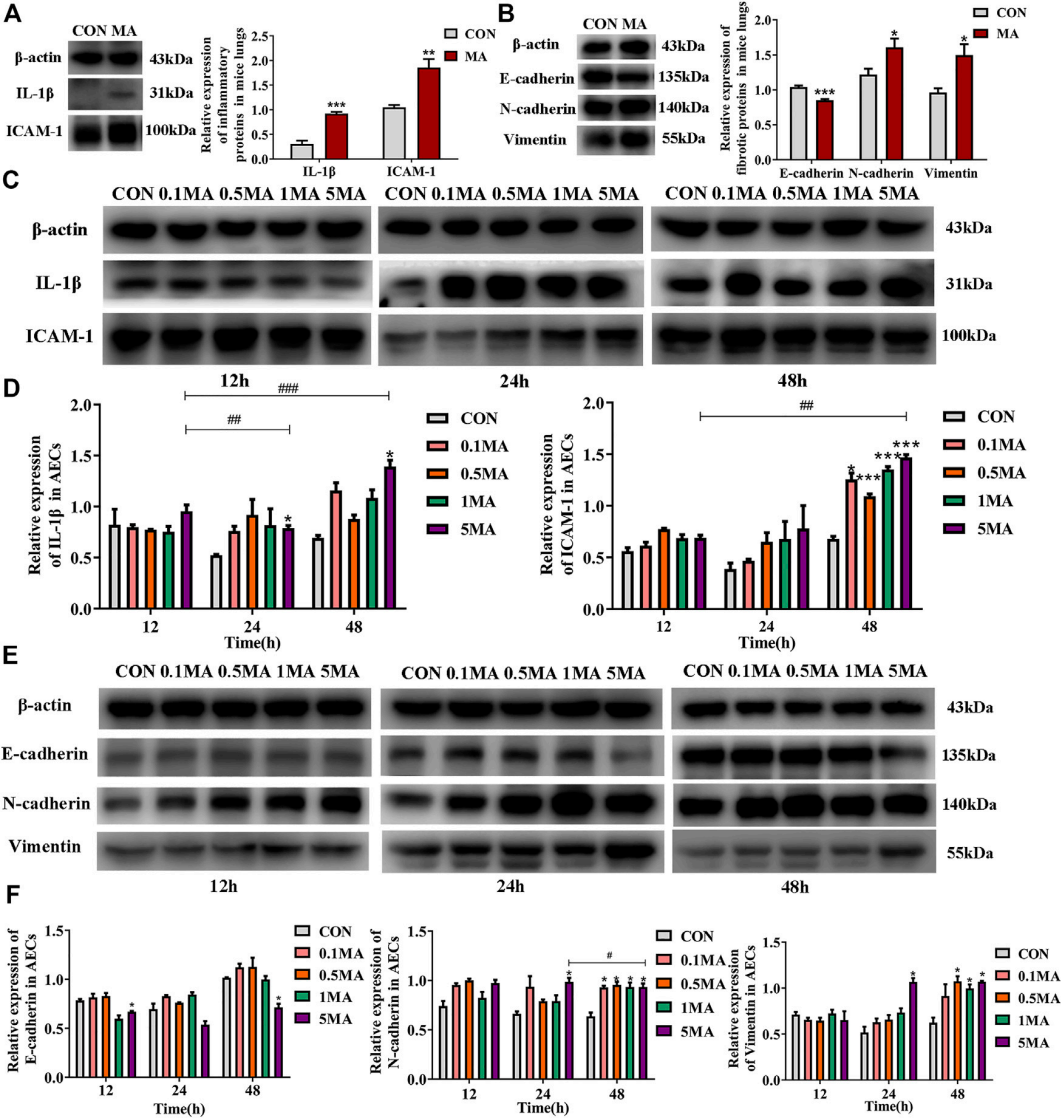
Inflammatory and fibrotic changes induced by MA. **(A)** The expression of IL-1β and ICAM-1 in the lungs of the mice in CON and the MA groups. N = 6. **(B)** The expression of E-cadherin, N-cadherin, and Vimentin in the lungs of the mice in the CON and the MA groups. N = 6. **(C)** Effects of MA exposure on IL-1β and ICAM expression in AECs. N = 3. **(D)** Relative expression of IL-1β and ICAM-1 in AECs. N = 3. € **(E)** Effects of MA exposure on E-cadherin, N-cadherin, and Vimentin expression in AECs. N = 3. **(F)** Relative expression of E-cadherin, N-cadherin, and Vimentin in AECs. N = 3. Data are expressed as mean ± standard deviation, ^*^ *p* < 0.05 vs. CON, ^**^ *p* < 0.01 vs. CON, ^***^ *p* < 0.001 vs. CON; ^##^ *p* < 0.01 vs. 5MA/12h, ^###^ *p* < 0.001 vs. 5MA/12h, ^#^ *p* < 0.05 vs. 5MA/24 h. AECs: Alveolar epithelial cells, CON: The control group, MA: The group treated with methamphetamine in mice, 0.1 MA: 0.1 mM methamphetamine, 0.5 MA: 0.5 mM methamphetamine, 1 MA: 1 mM methamphetamine, 5 MA: 5 mM methamphetamine.

An increase in expression of interleukin-1β (IL-1β) and intercellular adhesion molecule 1 (ICAM-1) was observed due to the chronic lung injury induced by MA exposure compared to the control (^**^ *p* < 0.01 vs. CON, ^***^ *p* < 0.001 vs. CON; Figure 3A). EMT biomarkers such as E-cadherin were significantly reduced, while the expression of N-cadherin and Vimentin were increased in the MA group (^*^ *p* < 0.05 vs. CON, ^***^ *p* < 0.001 vs. CON; Figure 3B) compared to the control group.

An increase in IL-1β and ICAM-1 expression in AECs was observed in a time and dosage-dependent manner (^*^ *p* < 0.05 vs. CON, ^##^ *p* < 0.01 vs. 5 MA/12h, ^###^ *p* < 0.001 vs. 5 MA/12h; ^*^ *p* < 0.05 vs. CON, ^***^ *p* < 0.001 vs. CON, ^##^ *p* < 0.01 vs. 5 MA/12h; Figures 3C,D). After treating AEC with MA, a significant decrease in the expression of E-cadherin was observed compared to the control group (^*^ *p* < 0.05 vs. CON, ^##^ *p* < 0.01 vs. 5 MA/12h; Figures 3E,F). However, there was a significant increase in the expression of N-cadherin in a time- and dosage-dependent manner on MA treatment in AEC compared to control (^*^ *p* < 0.05 vs. CON, ^#^ *p* < 0.05 vs. 5 MA/24 h; Figures 3E,F). The expression trend of Vimentin was similar to N-cadherin (^*^ *p* < 0.05 vs. CON; Figures 3E,F).

### 3.2 Effects of MA on RUNX3, ZEB1, and its downstream signaling molecules

**FIGURE 4:**
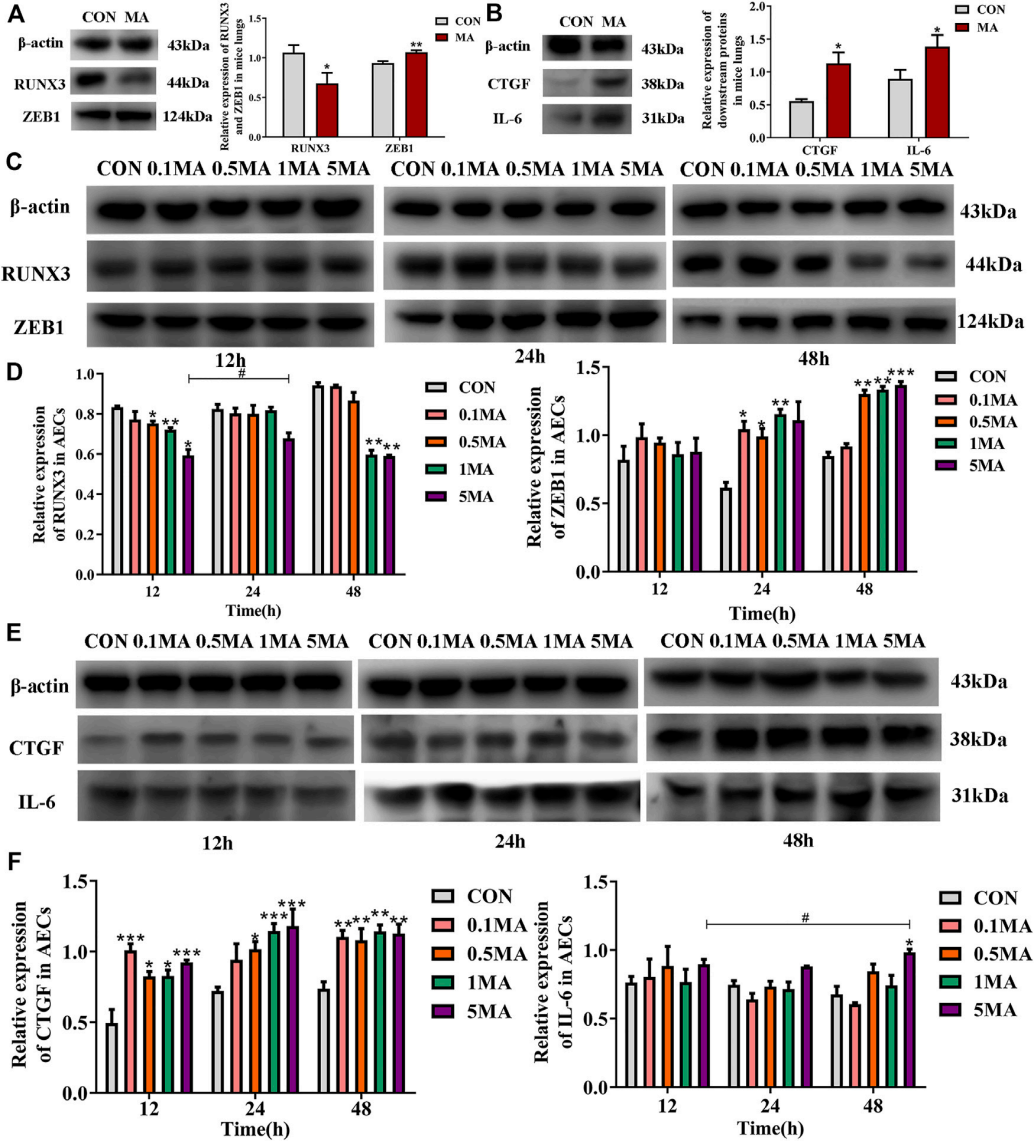
Effects of MA exposure on RUNX3, ZEB1 and its downstream signals. **(A)** RUNX3 and ZEB1 expression in mice lungs. **(B)** CTGF and IL-6 expression in mice lungs. **(C)** Effects of MA exposure on RUNX3 and ZEB1 expression in AECs. **(D)** Relative expression of RUNX3 and ZEB1 in AECs. **(E)** Effects of MA exposure on CTGF and IL-6 expression in AECs. **(F)** Relative expression of CTGF and IL-6 in AECs. N = 6 **(A**,**B)**, n = 3 **(C–F)**. Data are expressed as mean ± standard deviation, ^*^ *p* < 0.05 vs. CON, ^**^ *p* < 0.01 vs. CON, ^***^ *p* < 0.001 vs. CON; ^#^ *p* < 0.05 vs. 5MA/12 h. AECs: Alveolar epithelial cells, CON: The control group, MA: The group treated with methamphetamine in mice, 0.1 MA: 0.1 mM methamphetamine, 0.5 MA: 0.5 mM methamphetamine, 1 MA: 1 mM methamphetamine, 5 MA: 5 mM methamphetamine.

RUNX3 expression decreased in the mice lungs due to MA exposure (^*^ *p* < 0.05 vs. CON), while the ZEB1 expression increased (^**^ *p* < 0.01 vs. CON; Figure 4A) compared to the mice control group. The expression of the downstream signaling molecules like connective tissue growth factor (CTGF) was assessed. CTGF is an indicator of fibrosis and belongs to the hippo signaling pathway. CTGF expression was increased in the lungs of mice in the MA group compared to the mice in the control group (^*^ *p* < 0.05 vs. CON; Figure 4B). Interleukin-6 (IL-6) is an inflammatory cytokine and a key protein in the hippo signaling pathway. The expression of IL-6 was significantly increased in the lungs of mice in the MA group compared to the control group (^*^ *p* < 0.05 vs. CON; Figure 4B).

A significant reduction in RUNX3 expression in AECs was observed with an increase in MA dosage and exposure time (^*^ *p* < 0.05 vs. CON, ^**^ *p* < 0.01 vs. CON, ^#^ *p* < 0.05 vs. 5MA/12h; Figures 4C,D). A significant increase in ZEB1 expression on MA exposure in a time and dosage-dependent manner (^*^ *p* < 0.05 vs. CON, ^**^ *p* < 0.01 vs. CON, ^***^ *p* < 0.001 vs. CON; Figures 4C,D). An increase in expression of CTGF and IL-6 in AECs was observed with an increase in MA dosage and exposure time (^*^ *p* < 0.05 vs. CON, ^**^ *p* < 0.01 vs. CON, ^***^ *p* < 0.001 vs. CON; ^*^ *p* < 0.05 vs. CON, ^#^ *p* < 0.05 vs. 5MA/12h; Figures 4E,F).

### 3.3 Protein-protein interactions between RUNX3 and ZEB1

#### 3.3.1 Bio-informatic prediction of interactions between RUNX3 and ZEB1

**FIGURE 5:**
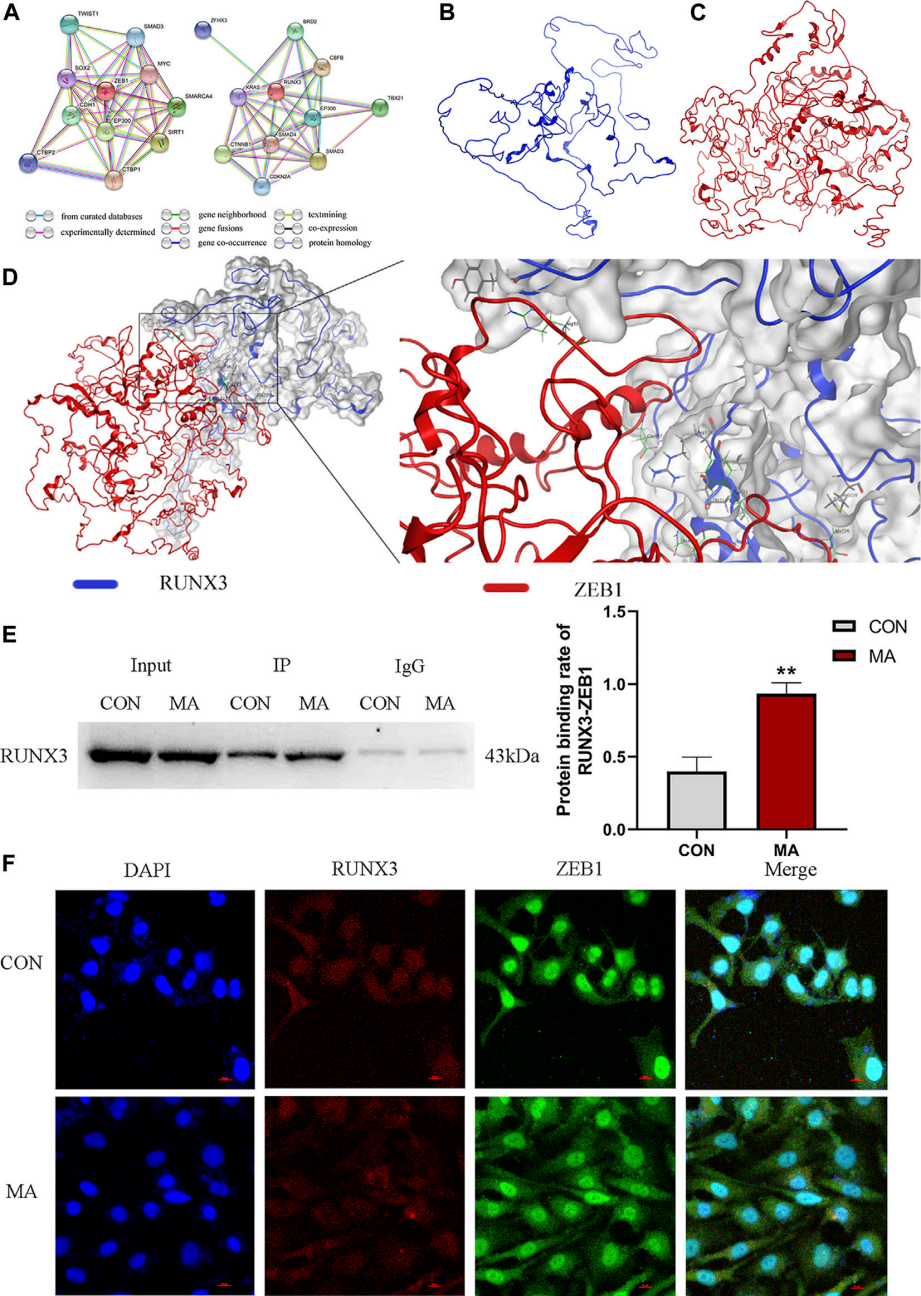
Protein-protein interactions (PPIs) between RUNX3 and ZEB1. **(A)** RUNX3-related gene network and ZEB1-related gene networks generated by STRING database. This network uses different color nodes to represent different proteins (ZFH3, BRD2, KRAS, RUNX3, CBFB, CTNNB1, SMAD4, EP300, TBX21, CDKN2A, SMAD3; TWIST1, SMAD3, SOX2, ZEB1, MYC, CDH1, EP300, SMARCA4, SIRT1, CTBP1, CTBP2). Different color lines represent different interactions at different gene levels (gene neighborhood, gene fusions, gene co-occurrence) or the protein levels (co-expression, protein homology). **(B)** The protein structure of RUNX3 predicted by the Robetta data bank. **(C)** The protein structure of ZEB1 predicted by the I-TASSER server. **(D)** Molecular docking of RUNX3 with ZEB1 using MOE software. MOE analysis showed the binding sites of the docking structure of RUNX3 to ZEB1. Blue ribbon: RUNX3; Red ribbon: ZEB1. **(E)** Co-IP assay for the PPIs between RUNX3 and ZEB1 in AECs and the protein binding rate of RUNX3-ZEB1. **(F)** Confocal immunofluorescence of RUNX3 and ZEB1 in AECs. N = 3. Data are expressed as mean ± standard deviation, ^**^ *p* < 0.01 vs. CON. CON: The control group, MA: The group treated with methamphetamine for 48 h. TWIST1: twist-related protein 1, SMAD3: small mother against decapentaplegic 3, SOX2: SRY-box transcription factor 2, ZEB1, Zinc finger E-box binding homeobox 1, MYC: Myelocytomatosis oncogene, CDH1, E-cadherin, EP300: E1A Binding Protein P300, SMARCA4: SWI/SNF-related, matrix-associated, actin-dependent regulator of chromatin, subfamily A, member 4, SIRT1: Silencing information regulator 2 related enzyme 1, CTBP1: C-terminal binding protein 1, CTBP2: C-terminal binding protein 2; ZFHX3: Zinc finger homeobox 3 gene, BRD2: bromodomain protein 2, KRAS: Kirsten rat sarcoma 2 viral oncogene homolog, RUNX3: Runt-related transcription factor 3, CBFB: Core binding factor beta, CTNNB1: cis-acting circRNA generated by beta-catenin, SMAD4: small mother against decapentaplegic 4, EP300: E1A Binding Protein P300, TBX21: T-box transcription factor protein 21, CDKN2A: Detection of homozygous deletion of the p16 gene, SMAD3: small mother against decapentaplegic 3. CON: The control group, MA: The group treated with methamphetamine for 48 h.

Based on the gene networks generated using the STRING database (*Homo sapiens*), the PPIs between RUNX3 and ZEB1 have not been reported (Figure 5A). The structure of RUNX3 was predicted using the Robetta server, and the structure of ZEB1 was predicted from the I-TASSER server (Figures 5B,C). The MOE software investigated the PPIs between RUNX3 and ZEB1 and bio-informatic prediction (Figure 5D). As shown in Table 3, there were five binding sites between RUNX3 and ZEB1. These results indicate that RUNX3 interacts with ZEB1.

**TABLE 3:** The docking results of RUNX3 and ZEB1.

Interaction type	RUNX3	ZEB1	Distance	E (kcal/mol)
Arene bond	Ser315	Tyr721	3.97	−0.6
Hydrogen bond	Met220	Ala726	4.02	−0.8
Hydrogen bond	Thr314	Asn715	3.08	−0.6
Hydrogen bond	Tyr348	Arg930	3.47	−1
Hydrogen bond	Arg316	Glu957	3.26	−1.1

#### 3.3.2 Identifying interactions between RUNX3 and ZEB1

The co-IP results confirmed that ZEB1 interacted with RUNX3. On MA exposure, the protein binding rate of RUNX3-ZEB1 increased compared to the control group in AECs (^**^ *p* < 0.01 vs. CON, Figure 5E).

The confocal immunofluorescence was performed to determine the cellular co-localization of RUNX3 and ZEB1. A decrease in RUNX3 expression on MA exposure and increased ZEB1 expression was observed. ZEB1 was primarily localized in the nucleus in the control group; however, in the MA group, the ZEB1 was localized both in the nucleus and the cytoplasm. RUNX3 was expressed in the cell nucleus and cytoplasm in the control and the MA groups. RUNX3 expression in cytoplasm was less in the MA group compared to the control group, while RUNX3 expression in nucleus remains the same. These results suggest a cellular co-localization of RUNX3 and ZEB1 (Figure 5F).

### 3.4 The interactions between RUNX3 and ZEB1 in MA-induced chronic lung injury

#### 3.4.1 Effects of interactions between RUNX3 and ZEB1 on cell viability

**FIGURE 6:**
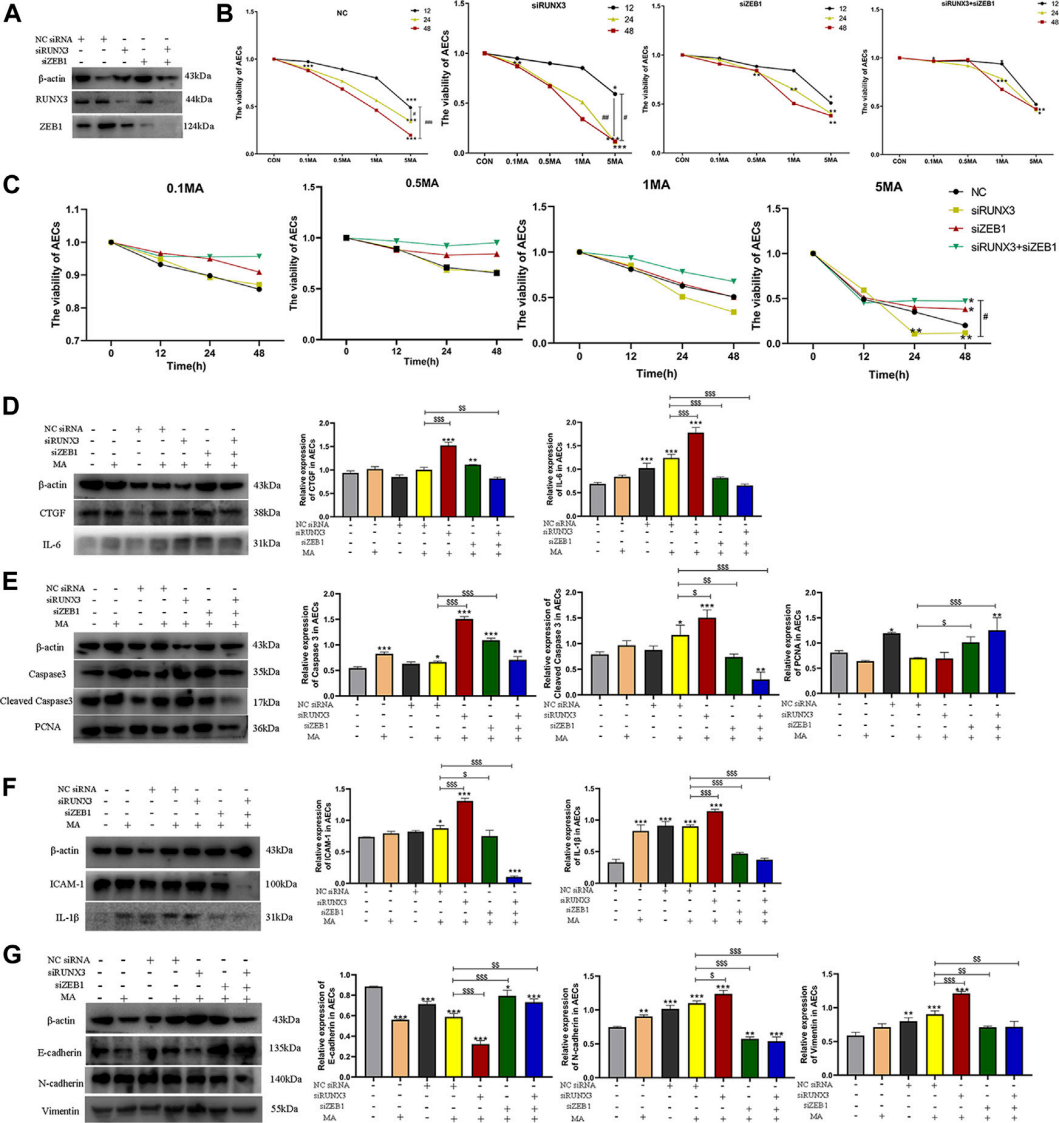
The interaction of RUNX3 with ZEB1 in MA-induced chronic lung injury. **(A)** The viability of AECs in different groups (NC, siRUNX3, siZEB1, siRUNX3+siZEB1) on MA exposure. **(B)** The viability of AECs on treatment with different dosages of MA. **(C)** Effects of MA exposure on RUNX3, ZEB1, CTGF, and IL-6 expression in AECs transfected with the corresponding siRNA. **(D)** Effects of MA exposure on caspase-3, cleaved caspase-3, and PCNA expression in AECs transfected with siRNA. **(E)** Effects of MA exposure on ICAM-1 and IL-1β expression in AECs transferred with siRNA. **(F)** Effects of MA exposure on E-cadherin, N-cadherin, and Vimentin expression in AECs with transfected siRNA. n = 3. Data are expressed as mean ± standard deviation, ^*^ *p* < 0.05 vs. CON, ^**^ *p* < 0.01 vs. CON, ^***^ *p* < 0.001 vs. CON; ^#^ *p* < 0.05 vs. 5MA/12h, ^##^ *p* < 0.01 vs. 5MA/12h; ^$^ *p* < 0.05 vs. NC + MA, ^$$^ *p* < 0.01 vs. NC + MA, ^$$$^ *p* < 0.001 vs. NC + MA. AECs: Alveolar epithelial cells, NC: negative control, MA: methamphetamine, siRUNX3: siRNA for the interference of RUNX3, siZEB1: siRNA for the interference of ZEB1, 0.1 MA: 0.1 mM methamphetamine, 0.5 MA: 0.5 mM methamphetamine, 1 MA: 1 mM methamphetamine, 5 MA: 5 mM methamphetamine.

siRUNX3, siZEB1, and siRUNX3+siZEB1 were successfully transfected in AEC and the transfection of siRNA was confirmed by western blots (Figure 6A). A change in the viability of AECs was observed in a time and dosage-dependent manner in different groups (Figure 6B). The viability of AECs in the siRUNX3+siZEB1 group was the highest, the viability of AECs in the siZEB1 group was higher compared to the Negative control (NC) group, The viability of AECs in the siRUNX3 group was the less compared to the NC group (Figure 6C). The CCK-8 assay results indicated that ZEB1 and the RUNX3-ZEB1 interactions reduced the viability of AECs, while RUNX3 expression enhanced the viability of AECs.

#### 3.4.2 The role of RUNX3, ZEB1, and their PPIs in the mechanism of chronic lung injury induced by MA

A significant decrease in CTGF and IL-6 expression was observed in the cells in the siRUNX3+siZEB1+MA group (^$$^ *p* < 0.01 vs. NC + MA; ^$$$^ *p* < 0.001 vs. NC + MA), whereas CTGF and IL-6 expression increased in the siRUNX3+MA group (^$$$^ *p* < 0.001 vs. NC + MA; ^$$$^ *p* < 0.001 vs. NC + MA; Figure 6D). The expression of CTGF was reduced in the siZEB1+MA group, and the expression of IL-6 was obviously decreased in the siZEB1+MA group (^$$$^ *p* < 0.001 vs. NC + MA; Figure 6D).

In the siRUNX3+siZEB1+MA group, a reduction in caspase-3 expression was observed. Further, a significant decrease in the expression of cleaved caspase-3 was observed in cells in the siRUNX3+siZEB1+MA group (^$$$^ *p* < 0.001 vs. NC + MA; Figure 6E). A significant increase in caspase-3 and cleaved caspase-3 expression was observed in cells in the siRUNX3+MA group (^$$$^ *p* < 0.01 vs. NC + MA; ^$^ *p* < 0.05 vs. NC + MA), whereas their expression significantly decreased in the siZEB1+MA group (^$$$^ *p* < 0.001 vs. NC + MA; ^$$^ *p* < 0.01 vs. NC + MA; Figure 6E). On the contrary, the expression of PCNA increased in the cells in the siRUNX3+siZEB1+MA group and the siZEB1+MA group (^$$$^ *p* < 0.001 vs. NC + MA; ^$^ *p* < 0.05 vs. NC + MA), PCNA expression decreased in the siRUNX3+MA group (Figure 6E).

The expression of ICAM-1 and IL-1β was significantly reduced in the cells in siRUNX3+siZEB1+MA and the siZEB1+MA groups (^$$$^ *p* < 0.001 vs. NC + MA; ^$$$^ *p* < 0.001 vs. NC + MA, ^$^ *p* < 0.05 vs. NC + MA; ^$$$^ *p* < 0.001 vs. NC + MA), and the expression of ICAM-1 and IL-1β significantly increased in the siRUNX3+MA group (^$$$^ *p* < 0.01 vs. NC + MA; ^$$$^ *p* < 0.01 vs. NC + MA; Figure 6F).

A significant reduction in E-cadherin expression was observed in the siRUNX3+siZEB1+MA and the siZEB1+MA groups (^$$^ *p* < 0.01 vs. NC + MA; ^$$$^ *p* < 0.001 vs. NC + MA); however, a significant increase in E-cadherin expression was observed in the siRUNX3+MA group (^$$$^ *p* < 0.001 vs. NC + MA; Figure 6G). The expression of N-cadherin and Vimentin was significantly reduced in the siRUNX3+siZEB1+MA group and the siZEB1+MA group (^$$$^ *p* < 0.001 vs. NC + MA; ^$$^ *p* < 0.01 vs. NC + MA, ^$$$^ *p* < 0.001 vs. NC + MA; ^$$^ *p* < 0.01 vs. NC + MA). Further, a significant increase in N-cadherin and Vimentin expression was observed in the siRUNX3+MA group (^$^ *p* < 0.05 vs. NC + MA; ^$$$^ *p* < 0.001 vs. NC + MA; Figure 6G).

## 4 Discussion

MA is a major substance abuse drug in adults (Jones et al., 2020). MA abuse damages the organs and causes apoptosis and lesions in the respiratory system (Gu et al., 2017; Kevil et al., 2019; Wang et al., 2020). In this study, we show that MA causes alveolar injury and hemodynamic changes. MA exposure also leads to AECs apoptosis, inflammation, and fibrosis of the lungs. It also decreases RUNX3 expression and increases the expression of ZEB1 and affect the expression of their downstream signaling molecules. Our results revealed that the interactions between RUNX3-ZEB1 exist, and the protein binding rate between RUNX3 and ZEB1 increases on MA exposure.

RUNX3 and ZEB1 are two important proteins in metabolism. DNA damage stimulates the TP53 expression to inhibit RUNX3 expression in cancer (Date and Ito, 2020). MiR-301a suppresses RUNX3 expression, which accelerates the progression of lung cancer (Li X et al., 2019). In pulmonary diseases, RUNX3 induces the apoptosis of airway epithelial cells on viral infections (Gan et al., 2015). Previous studies have shown an association between RUNX3 and MA-induced EMT (Shi et al., 2020). Mounting evidence has demonstrated that ZEB1 is an important mediator of EMT, causes fibrosis, and damages various organs (Lee et al., 2018; Qian et al., 2019; Yuan et al., 2020). ZEB1, along with E-cadherin, are essential for fibrosis in AECs (Zhang X et al., 2020). In animal models, chronic exposure to MA causes an apparent change in cardiopulmonary functions and pulmonary parenchymal lesions. MA exposure suppressed RUNX3 and increased ZEB1 expression to facilitate MA-induced lung injury. Various studies have reported that CTGF and IL-6 are the downstream signaling molecules of RUNX3-ZEB1, which play an important role in promoting fibrosis and inflammation (Zhao et al., 2008; Lehmann et al., 2016; Kulkarni et al., 2018; Zhou X et al., 2019). As expected, the increase in expression of the downstream signaling molecules (CTGF, IL-6) was observed in MA-induced chronic lung injury.

Mechanisms associated with MA-induced lung injury is various. For example, MA-induced lung injury could be attributed to nuclear factor erythroid-2-related factor 2 (Nrf2)-mediated antioxidative defense (Bai et al., 2017). Interestingly, PPIs in MA-induced chronic lung injury have never been reported. However, PPIs play an important role in lung diseases. Tribble’s homolog 3/glycogen synthase kinase-3β (TRIB3‒GSK-3β) interactions promote lung fibrosis and could be used as the potential therapeutic target (Liu et al., 2021). The PPIs between Kelch-like ECH-associated protein 1 (Keap1) and Nrf2 play a critical role in acute lung injury (Wan et al., 2020). Based on this evidence, MOE software was used to predict the PPIs between RUNX3 and ZEB1. Co-IP assay and confocal immunofluorescence assay identified the interactions between RUNX3-ZEB1 in MA-induced chronic lung injury. Previous studies show that RUNX3 and ZEB1 function as transcription factor s in the cell nucleus, consistent with our results (Sánchez-Tilló et al., 2011; Chen et al., 2016; Zhou W. N et al., 2019; Mevel et al., 2019; Guo et al., 2022). The co-IP results reveal an increase in the protein binding rate of RUNX3-ZEB1 on MA exposure, which indicated the increase in PPIs between RUNX3 and ZEB1 in MA-induced chronic lung injury.

RUNX3 and ZEB1 expression was knockdown in AECs to study the role of RUNX3, ZEB1, and the RUNX3-ZEB1 interactions. Inflammation, fibrosis, AECs apoptosis, and the increased expression of the downstream signals were alleviated by the knockdown of ZEB1 and the restraint of RUNX3-ZEB1 interactions in MA-induced lung injury. These pathological changes were aggravated by RUNX3 knockdown and MA exposure. RUNX3 protection against MA-induced lung injury was reduced due to its interactions with ZEB1. Further, ZEB1 expression is detrimental to MA-induced lung injury.

In this study, we explored the mechanism of MA-induced chronic lung injury from the perspective of PPIs, and confirmed that the RUNX3-ZEB1 interactions are critical in this mechanism. Recently, PPIs have been used in clinical settings. In 2017, FDA approved Venetoclax, a novel BCL-2 inhibitor targeting the PPIs between BCL-2 and other proteins in acute myeloid leukemia treatment (DiNardo et al., 2019; Guerra et al., 2019). Currently, various inhibitors targeting PPIs are under clinical trials, such as APG-1252 targeting Bcl-2/Bcl-XL (B-cell lymphoma-extra-large) interactions for anti-tumor therapy (Yi et al., 2020; Luo et al., 2021), and APG-115 targeting MDM2 (Mouse double minute 2 homolog)-p53 interactions in anti-tumor therapy (Fang et al., 2019; Fang et al., 2021). Results from this study suggest that RUNX3-ZEB1 interactions could be detrimental to MA-induced lung injury. These results demonstrated that RUNX3, ZEB1, and RUNX3-ZEB1 interactions play an important role in MA-induced chronic lung injury.

In conclusion, lung injury is caused by chronic exposure to MA. MA regulated RUNX3, ZEB1, and their downstream signaling molecules. Our results confirmed the interactions between RUNX3 and ZEB1. RUNX3-ZEB1interactions affect AECs viability and are involved in MA-induced chronic lung injury. Therefore, blocking RUNX3-ZEB1 interactions can alleviate the MA-induced chronic lung injury, which could serve as a novel therapeutic strategy for the chronic lung injury induced by MA.

## Data Availability

The raw data supporting the conclusions of this article will be made available by the authors, without undue reservation.
